# Hydrophobic Properties of Pine Wood Coatings Based on Epoxy Varnish and (Fluoro)Alkyl Methacrylate Copolymers

**DOI:** 10.3390/polym17233172

**Published:** 2025-11-28

**Authors:** Viktor V. Klimov, Vladislav V. Arkhipov, Olga V. Klimova, Manh D. Le, Evgeny V. Bryuzgin, Alexander V. Navrotskii

**Affiliations:** 1 Chemical Engineering Faculty, Volgograd State Technical University, 28 Lenin Ave, 400005 Volgograd, Russia; 2 Southern Branch of Joint Vietnam-Russia Tropical Science and Technology Research Center, 3, 3/2 Str., District Vuon Lai, Ho Chi Minh City 740300, Vietnam

**Keywords:** water-repellent coatings, modification, pine wood, epoxy varnish, methacrylic copolymers, superhydrophobicity

## Abstract

This study presents water-repellent coatings for pine wood surfaces based on epoxy varnish modified with glycidyl methacrylate–(fluoro)alkyl methacrylate copolymers, achieving superhydrophobic properties with contact angles up to 155° while maintaining the natural texture of the wood. The influence of the application method on the microtexture and water-repellent properties of the coatings has been demonstrated. Incorporating functional copolymers considerably improves water resistance: after 60 days of immersion, water absorption is reduced more than threefold compared to coatings made with unmodified epoxy varnish. Furthermore, the coatings maintain their water-repellent properties and preserve the wood’s appearance even after six months of exposure to the tropical climate of South Vietnam.

## 1. Introduction

Currently, wood is increasingly used in construction engineering owing to its natural origin and aesthetic appeal, finding applications in flooring, wall finishes, furniture, and even wooden bathtubs and sinks [1]. Of note, wood is a water-absorbing material [2,3]; repeated cycles of moisture sorption and desorption considerably reduce the microbiological stability of wooden products [4], and a high moisture content contributes to the appearance of black spots and mold. According to a previous study [5], a moisture content of 20 wt.% is sufficient for a wooden product to start decaying. Owing to the anisotropic properties of wood in the longitudinal, radial, and tangential directions, water absorption leads to varying degrees of swelling along these axes [6,7]. Therefore, the high hygroscopicity and low dimensional stability of wood cause critical defects during use, including warping [8], cracks [9], and deformations [10], which limit wider application and long-term operation in building structures under atmospheric conditions [11].

Superhydrophobic treatment of wood offers considerable potential for enhancing water and dust resistance, improving dimensional stability, and extending service life [12,13]. To impart highly hydrophobic and superhydrophobic properties to wood surfaces, various coating techniques have been reported, including immersion [14], spraying [15], hydrothermal synthesis [16], sol–gel processes [17], and graft polymerization [18], among others. From a practical perspective, immersion and spraying are particularly attractive because they avoid multi-step procedures and do not require additional equipment. Of note, the literature analysis in the review [19] clearly shows that, despite the variety of methods for producing superhydrophobic coatings, most studies focus on combining micro- and nanoparticles (such as silicon dioxide, titanium dioxide, and aluminum oxide) with (fluoro)silanes, long-chain fatty acids, or perfluorinated acids to lower surface free energy.

Currently, polymers are the most widely used materials for superhydrophobic coatings, functioning both as matrices and additives. Their popularity stems from availability, low cost, and advanced synthetic capabilities, which enable the production of functional copolymers with diverse architectures and the introduction of new properties [20]. Traditionally, barrier coatings protect substrate surfaces by providing passive moisture resistance, with degradation typically occurring at defects formed during application or service. Particularly noteworthy is the development of paints and composite coatings based on natural compounds and commercially available polymer matrices with functional additives [21]. In this approach, the use of low- or high-molecular-weight additives is essential to reduce surface free energy [22]. Importantly, this strategy also provides active protection against moisture penetration by creating highly hydrophobic and superhydrophobic states on the coating surface.

The traditional approach for surface protection involves applying coatings based on natural film-forming agents. Common bases for such coatings include oils, waxes, and paint or varnish materials derived from synthetic polymers. Natural oils such as soy, linseed, and tung [23,24,25] and waxes [26,27] are widely used as protective coatings that impart water-repellent properties and slow down microbiological degradation. Such coatings primarily serve a decorative purpose, enhancing the aesthetic qualities of wooden products; however, they do not always provide complete protection against external factors, which can lead to material degradation.

Paint coatings based on synthetic film-forming agents form thicker protective films that insulate wood from moisture and microbial attack, thereby enhancing durability under various operating conditions [28]. Such coatings are typically produced through thermal or chemical modification [29] or vacuum impregnation [30]. Among these, epoxy binders have received particular attention owing to their outstanding mechanical properties [31], thermal and chemical resistance [32], and excellent adhesion to various types of substrates [33]. However, coatings formulated with unmodified epoxy binders are inherently hydrophilic and susceptible to degradation under natural conditions [20]. Therefore, achieving high or even superhydrophobic performance while retaining the desirable properties of epoxy resin-based coatings is particularly relevant [34]. Thus, in a previous study [35], polytetrafluoroethylene additives were incorporated into an epoxy matrix to produce superhydrophobic coatings with contact angles of up to 159°. An alternative approach is described in the work of Kuzina [36], where industrial epoxy enamel EP-140 was used as the base material, subsequently textured and treated with perfluorosilane. The results of these studies demonstrate the high mechanical resistance of epoxy enamel, confirming its potential for long-term use in environments with high humidity. For instance, in a previous work [37], a nanocomposite coating composed of aminofunctionalized polydimethylsiloxane and epoxy resin was applied to wood, achieving a superhydrophobic state with contact angles of up to 165°. Of note, the scientific literature includes numerous studies on the hydrophobization of wood surfaces using formulations based on natural or synthetic polymers, most commonly involving oils, waxes, silanes, and phenol–formaldehyde compositions [38,39,40,41]. However, reports on the application of epoxy resins as wood coatings remain limited.

In our previous studies [42,43], we developed reactive copolymers based on glycidyl methacrylate (GMA) and 2,2,3,3,4,4-heptafluorobutyl methacrylate (HFBMA) or stearyl methacrylate (SMA) as effective agents for lowering surface free energy. These works demonstrated successful modification of wood surfaces through impregnation with copolymer solutions in methyl ethyl ketone (MEK) [44] and linseed oil [45], achieving superhydrophobic properties with contact angles of up to 167°. In this study, we propose water-repellent composite coatings based on industrial epoxy varnish EP-2146 combined with functional methacrylic copolymers. These copolymers possess reactive epoxy groups that ensure compatibility with the epoxy varnish, along with SMA and HFBMA substituents that reduce surface free energy. Following the principles of “green chemistry,” which aim to eliminate or minimize the use of fluorinated compounds and to improve copolymer compatibility with the epoxy varnish matrix, we synthesized a new ternary copolymer, poly(GMA-co-HFBMA-co-SMA), with reduced fluorinated comonomer content. The aim of this study is to investigate the formation of coatings on pine wood surfaces using epoxy varnish modified with functional GMA and (fluoro)alkyl methacrylate copolymer additives, and to evaluate the stability of their hydrophobic properties and durability under tropical climate conditions.

## 2. Materials and Methods

### 2.1. Materials

Pine wood was used in the form of samples with dimensions of 20 (radial) × 20 (tangential) × 10 (longitudinal) mm, in accordance with national standard [46]. The following reagents and materials were used: analytical-grade solvents—MEK (99%, Vekton, St Petersburg, Russia), n-hexane (99%, Vekton, Russia), methanol (99.5%, Vekton, Russia) and distilled water; epoxy parquet varnish (EP-2146) produced by Yaroslavl Paints LLC [47]. Monomers and initiators included GMA (97%), HFBMA (99.5%), SMA (97%), and azobisisobutyronitrile (AIBN, 98%) (all supplied by Aldrich, Milwaukee, WI, USA). Before use, GMA was purified by vacuum distillation at 50 °C.

### 2.2. Synthesis of GMA and Alkyl Methacrylate/Fluoroalkyl Methacrylate (AlMA/FMA) Copolymers

Statistical copolymers of GMA and AlMA/FMA were synthesized in MEK at 70 °C for 24 h using AIBN as the initiator. The total monomer concentration was 1 mol/L. The molar ratios of monomers were as follows: [GMA]:[AlMA] = 1.5:1; [GMA]:[FMA] = 0.5:1; and [GMA]:[FMA]:[AlMA] = 1:2:1. A detailed description of the synthesis procedure is provided in a previous study [45].

### 2.3. Preparation of Impregnation Solutions

Solutions of binary copolymers poly(GMA-co-SMA) and ternary copolymers poly(GMA-co-HFBMA-co-SMA) were prepared using epoxy varnish as the solvent. A 5 wt.% suspension of the copolymer was dissolved in epoxy varnish at 70 °C under stirring for 60 min.

For the preparation of poly(GMA-co-HFBMA) copolymer solutions, a mixed solvent system (epoxy varnish and MEK) was used. First, 5 wt.% solutions of poly(GMA-co-HFBMA) were obtained in MEK according to the procedure described in a previous study [36]. Epoxy varnish was then added to these solutions at a weight ratio of epoxy varnish:MEK = 1:1, followed by stirring at 70 °C for 30 min.

### 2.4. Modification of Pine Wood Samples by Immersion in Impregnation Solutions

Wood samples were pre-washed in an ultrasonic bath with deionized water for 15 min and then dried according to national standard GOST 16483.7-71 [48]. The dried samples were placed individually in separate containers to avoid contact. To prevent flotation during impregnation, the samples were covered with a mesh, and a weight was applied on top. Then, 5 wt.% copolymer solutions were poured into the containers, ensuring complete immersion of the samples and maintaining a solution layer of at least 5 mm above the wood surface. Impregnation was performed for 2 h. Afterward, the samples were removed from the solutions, transferred to clean containers, and allowed to dry at room temperature for 24 h.

### 2.5. Modification of Pine Wood Samples by Brush Application

Preliminary sample preparation was performed as described above. A composite coating was then applied to all edges of the dried wood samples using a brush. After the initial application, the samples were left to air-dry at room temperature for 2 h. A second layer of the coating was subsequently applied, and the samples were allowed to dry at room temperature for an additional 24 h. This procedure was performed according to the manufacturer’s instructions.

### 2.6. Methods

The molecular weight characteristics of the polymers were determined by gel permeation chromatography (GPC) using a Shimadzu system (Tokyo, Japan) equipped with columns packed with polystyrene gel (pore sizes of 1 × 10^5^ and 1 × 10^4^ Å). Tetrahydrofuran (THF) was used as the eluent at 40 °C, and a differential refractometer served as the detector. Chromatograms were analyzed using LCsolution software (version 1.25), and highly dispersed polymethylmethacrylate (PMMA) standards were used for calibration.

The morphology of the coating surfaces was examined using scanning electron microscopy (SEM) on a Versa 3D instrument (FEI, Morristown, NJ, USA) in low-vacuum mode with chamber water vapor pressure of 10–80 Pa, accelerating voltages of 10–20 kV, and beam currents of 13 pA to 4 nA. The elemental composition of the surfaces was determined by energy-dispersive X-ray spectroscopy (EDS) using an Oxford 51N1286 AZtecLive Expert system equipped with an Ultim Max 65 detector (Oxford Instruments, Abingdon, UK).

Infrared (IR) spectra of the sample surfaces were recorded using an FT-801 Fourier-transform infrared (FTIR) spectrometer (SIMEX, Moscow, Russia) over the range of 450–4000 cm^−1^. Spectra were acquired using single-bounce attenuated total reflection (ATR) and diffuse reflectance infrared Fourier transform spectroscopy (DRIFTS) techniques on a universal attachment equipped with a zinc selenide (ZnSe) crystal and a DRIFTS insert.

The water contact angle was measured using an OCA 15 EC system (DataPhysics, Filderstadt, Germany). Drops of deionized water (5–7 µL) were applied to the sample surface, and the contact angle of the sessile droplet was determined using the Young–Laplace method. Measurements were performed at 6–8 locations on both surfaces of each sample, and the arithmetic mean of the contact angles was calculated.

Dynamic studies of droplet behavior on the surfaces of modified samples over extended time intervals were performed in a water vapor-saturated chamber. Under high-humidity conditions and without exposure to the external environment, the slow evaporation of droplets allowed the monitoring of changes in the contact angle of sessile droplets over extended periods. Contact angle measurements were performed following the procedure described above.

Water absorption of the wood samples was evaluated in accordance with national standard GOST 21523.5-77 [49]. The modified samples were placed in a desiccator under an insert and then partially immersed in distilled water, leaving one cross-sectional surface dry. The desiccator was covered with a lid and maintained at 20 °C ± 2 °C. To monitor water absorption, the samples were periodically removed, their surfaces gently blotted with filter paper, and weighed. The first measurement was taken 2 h after immersion, followed by subsequent measurements at 1, 2, 3, 6, 9, 13, and 20 days, and thereafter every 10 days. The test was concluded when the difference between two consecutive weighings was ≤0.05 g.

Climatic tests were performed in the tropical environment of South Vietnam at the Dam Bai Marine Testing Station (Nha Trang). Samples were mounted on experimental stands facing south with an inclination of 45° to the horizon and exposed for 6 months. Visual inspections and contact angle measurements were performed monthly.

## 3. Results

The contemporary “green chemistry” approach advocates eliminating or minimizing the use of fluorine-containing compounds. However, in the development of superhydrophobic coatings, fluorinated low- and high-molecular-weight modifiers are notable for their exceptionally low surface free energy values [50]. The previously described reactive copolymers based on GMA and HFBMA/SMA were used as modifiers. These copolymers were chosen for their affinity for epoxy varnish, allowing the varnish to serve as a solvent-free base. However, when using the poly(GMA-co-HFBMA) copolymer, a heterogeneous system forms, requiring the use of a mixed solvent (epoxy varnish/MEK) to achieve proper dissolution. To address this limitation and the reduced fluorinated comonomer content, we additionally synthesized ternary copolymers, poly(GMA-co-HFBMA-co-SMA), in which the incorporation of stearyl methacrylate increases the copolymer’s affinity for industrial epoxy varnish, enabling complete dissolution without the use of organic solvents. The synthesized ternary copolymer exhibits a low molecular weight (M_n_ = 7.7 × 10^4^; M_w_ = 12.6 × 10^4^) and a relatively narrow molecular weight distribution, with a polydispersity index of 1.6. This allows for direct comparison of the properties of polymer coatings depending on the structure of the functional comonomer.

Coatings based on modified epoxy varnish were applied using two methods: impregnation, where wood samples were fully immersed in the composition, and brush application, following the manufacturer’s recommendations. The adhesion of the composite coatings on the wood surface was monitored gravimetrically. Table 1 shows that the application method had little effect on the overall mass of the coatings, with an average weight gain of approximately 17–20 wt.%. The smallest weight increase was observed for coatings based on poly(GMA-co-HFBMA) prepared from a mixed solvent, with weight gains of 10–17 wt.%. Of note, the application method can influence coating properties: impregnation promotes penetration of the modifying solution into the open pore structure of the wood, whereas brush application primarily forms a surface film.

**Figure 1:**
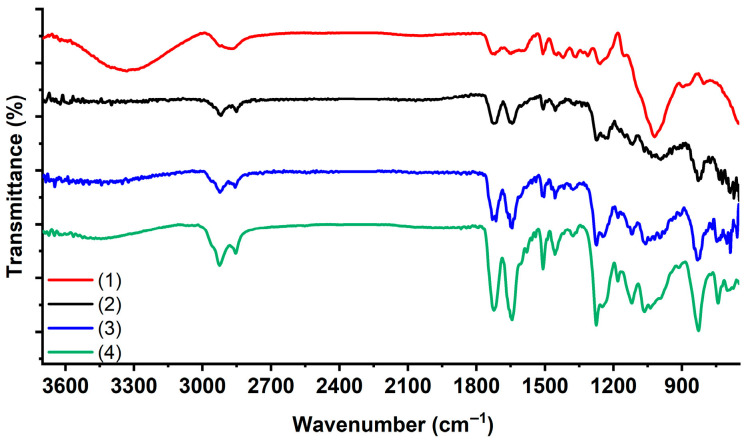
FTIR spectra of wood samples modified with the base epoxy varnish (1) and varnish containing copolymer additives: (2) poly(GMA-co-HFBMA), (3) poly(GMA-co-SMA), (4) poly(GMA-co-HFBMA-co-SMA).

The adhesion of copolymers to the wood surface was confirmed by FTIR spectroscopy (Figure 1). In the spectra of wood modified with GMA and HFBMA/SMA copolymers, characteristic absorption bands appear at 2857 and 2923 cm^−1^, corresponding to the stretching vibrations of methylene and methyl groups. Additionally, the intensity of the carbonyl band at 1746 cm^−1^ increases, accompanied by a shift to 1725–1735 cm^−1^, indicative of the methacrylate carbonyl groups. In the spectrum of unmodified wood, the peak at 1248 cm^−1^ is assigned to C–O stretching vibrations in lignin, while the peak at 1263 cm^−1^ corresponds to C–O stretching in simple ether bonds. As a result of the modification, additional spectral changes are observed. For wood treated with the poly(GMA-co-HFBMA) copolymer, a prominent peak appears at 1223 cm^−1^, corresponding to C–F stretching vibrations. Furthermore, bands associated with the asymmetric stretching of epoxy rings are observed in the 905–908 cm^−1^ range, along with deformation vibrations of the C–O–C bond of the oxirane ring near 831 cm^−1^.

**Figure 2:**
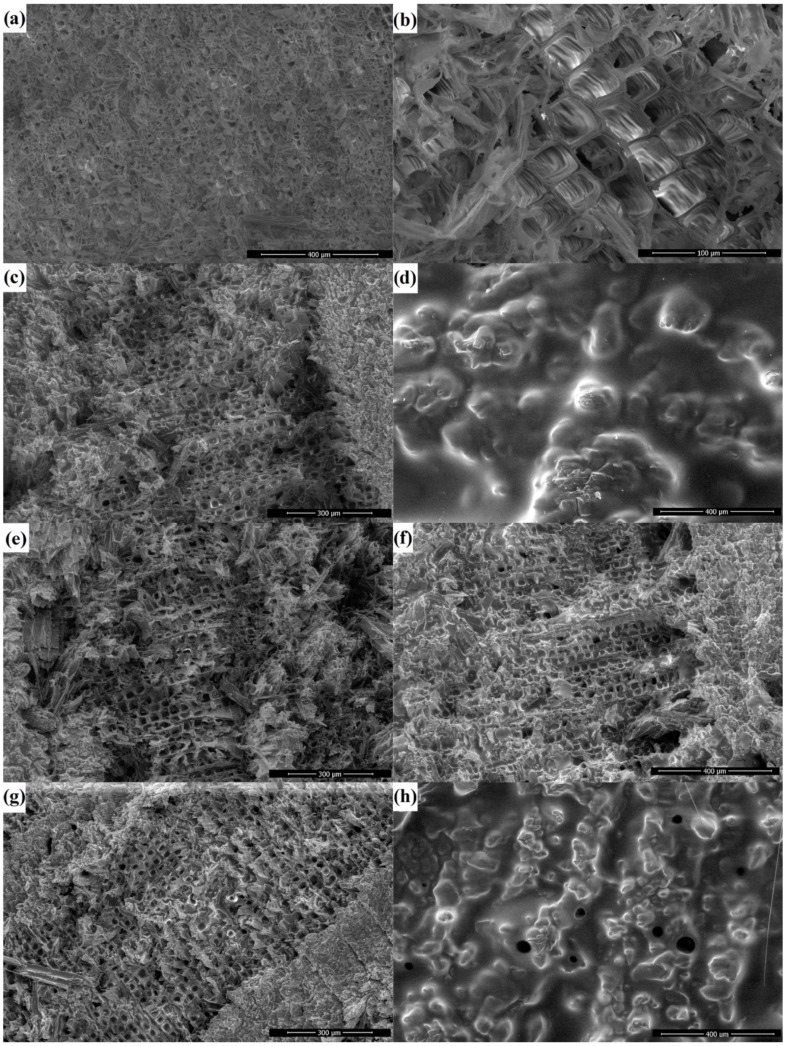
SEM images of the transverse surface of wood: unmodified wood (**a**) ×300; (**b**) ×2400; modified with epoxy varnish applied by immersion (**c**) ×300 and by brush (**d**) ×300; epoxy varnish with poly(GMA-co-HFBMA) additive applied by immersion (**e**) ×300 and by brush (**f**) ×300; epoxy varnish with poly(GMA-co-HFBMA-co-SMA) additive applied by immersion (**g**) ×300 and by brush (**h**) ×300.

SEM images (Figure 2) reveal changes in surface morphology after epoxy varnish modification of wood, with the method of coating application playing a considerable role. Brush application (Figure 2d,h) produces a continuous and relatively thick coating, resulting in the smoothing of the original microtexture of the wood surface. In contrast, immersion-based application (Figure 2c,g) preserves the original microtexture, while forming a thin film that partially covers the open pore structure of the wood. Of note, coatings based on epoxy varnish containing poly(GMA-co-HFBMA) were prepared using a mixed solvent system with MEK, which facilitates copolymer dissolution and reduces the formulation viscosity. Therefore, thinner coatings are formed (Figure 2d,e). Even when applied with a brush, the resulting film is relatively thin and only partially covers the wood’s pore structure. The reduced viscosity enables improved penetration of the composite coating into the wood matrix, potentially enhancing water-repellent properties. However, it is also noteworthy that, even for thicker brush-applied coatings, defective areas are observed on the surface (Figure S1 in Supplementary Materials), likely owing to the application method. These defects may act as potential sites for degradation upon exposure to water or aggressive media.

The chemical composition of the near-surface layer was analyzed using EDS. As shown in Table 2, the formation of coatings—both with unmodified epoxy varnish and with copolymer-modified formulations—results in an increase in carbon content of up to 10 at.% compared with uncoated wood. Brush application of the coatings results in a further increase in carbon concentration, indicating the formation of a thicker layer that partially shields the native wood surface’s oxygen-containing functional groups. When copolymers containing fluorinated substituents are incorporated, the presence of fluorine is detected on the wood surface. Notably, for samples modified with the epoxy varnish/MEK–poly(GMA-co-HFBMA) composition, a decrease in surface carbon concentration is observed. This reduction is attributed to the use of the mixed solvent, which lowers the viscosity and results in thinner coatings, consistent with the gravimetric measurements.

As previously shown [44], the surface of unmodified, dried wood initially exhibits a highly hydrophobic state, with water contact angles up to 121°. However, water droplets are completely absorbed in less than a minute owing to the presence of accessible polar, oxygen-containing groups in the wood’s chemical structure. Modification of the wood surface with epoxy varnish, in the absence of functional copolymers, produces a coating that provides passive protection against water penetration. While this treatment considerably slows water absorption, it does not substantially increase contact angles because unshielded oxygen-containing groups remain exposed on the film surface.

It is noteworthy that the hierarchical structure revealed by a transverse wood cut is sufficient to achieve a superhydrophobic state, provided a surface energy-reducing agent is applied. For example, when a copolymer solution in an organic solvent is used as a modifier, a thin film forms without changing the surface microtexture, yet superhydrophobic properties are attained [44]. In this study, a wood surface with similar roughness parameters served as the substrate. The coating application method modifies the surface morphology and, therefore, affects the wetting characteristics [51,52]. The incorporation of functional copolymers based on AlMA/FMA and GMA into the epoxy varnish considerably increases water contact angles. The method of coating application also strongly influences wettability. As noted above, brush application produces thicker coatings that smooth the original microtexture of the wood surface, resulting in a hydrophobic state with contact angles only up to 110° (Table 3). In contrast, immersion of wood samples in the modifying solution promotes deeper penetration and preserves surface microtexture, achieving a highly hydrophobic state with contact angles up to 138°. Of note, these high contact angles are observed for coatings containing poly(HFBMA-co-SMA-co-GMA) ternary copolymers prepared in formulations without additional organic solvents. The highest water-repellent performance is achieved with the addition of the poly(HFBMA-co-GMA) copolymer, which is attributed to the reduced viscosity of the modifying system. Coatings formed by immersion of wood samples in the modifier solution exhibit superhydrophobic behavior, with contact angles reaching up to 155°. In contrast, brush-applied coatings achieve a highly hydrophobic state, with contact angles up to 138°.

**Figure 3:**
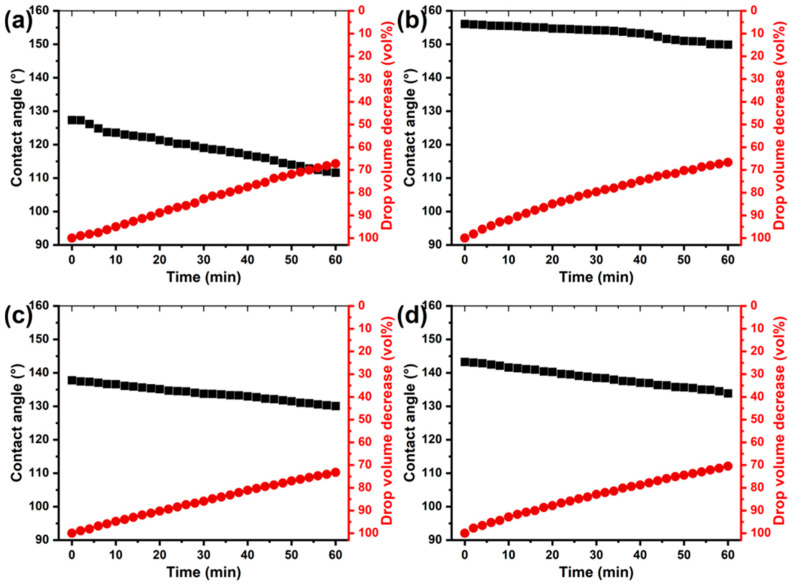
Dependence of the contact angle and volume (vol.%) of a water droplet under saturated conditions on contact time for wood surfaces modified by immersion in the base epoxy varnish (**a**) and varnish containing copolymer additives: poly(GMA-co-HFBMA) (**b**), poly(GMA-co-SMA) (**c**), and poly(GMA-co-HFBMA-co-SMA) (**d**).

**Figure 4:**
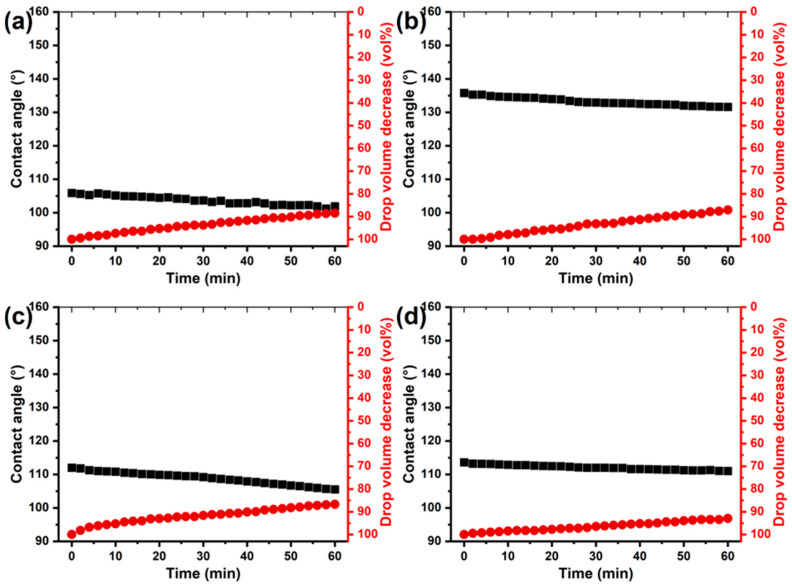
Dependence of the contact angle and volume (vol.%) of a water droplet under saturated conditions on contact time for wood surfaces coated by brush with the base epoxy varnish (**a**) and varnish containing copolymer additives: poly(GMA-co-HFBMA) (**b**), poly(GMA-co-SMA) (**c**), and poly(GMA-co-HFBMA-co-SMA) (**d**).

It should be noted that the initial contact angles reflect only the surface’s interfacial properties, and it is essential to evaluate the long-term stability of this state. To assess the stability of the hydrophobic properties of the coatings, changes in both the contact angle and the volume of water droplets were monitored over time during prolonged contact with the sample surface. Measurements were performed in a closed chamber saturated with water vapor to prevent droplet evaporation [53]. Figure 3 and Figure 4 show that, for all coatings, contact angle and droplet volume change over time, regardless of application method or hydrophobic modifier type. However, coatings applied by immersion, despite exhibiting higher initial contact angles, show a more pronounced decrease in wettability and a higher rate of water absorption after 1 h of droplet contact. The addition of copolymers to the varnish increases the stability of the coatings: the decrease in contact angle is limited to approximately 10°, compared with 18° for coatings with unmodified varnish. However, the copolymers have little effect on the rate of droplet absorption, with a volume reduction of approximately 30 wt.%.

Brush application produces thicker coatings, which increases passive protection against moisture, as evidenced by a more than twofold reduction in the rate of water absorption for all coating types (Figure 4). Additionally, the hydrophobic state is more stable, with contact angle changes of only 3–11° after 1 h of water droplet contact. The best performance is observed for immersion-applied coatings containing the poly(GMA-co-HFBMA) copolymer, which maintain a superhydrophobic state with contact angles of up to 150° after 1 h (Figure 3).

**Figure 5:**
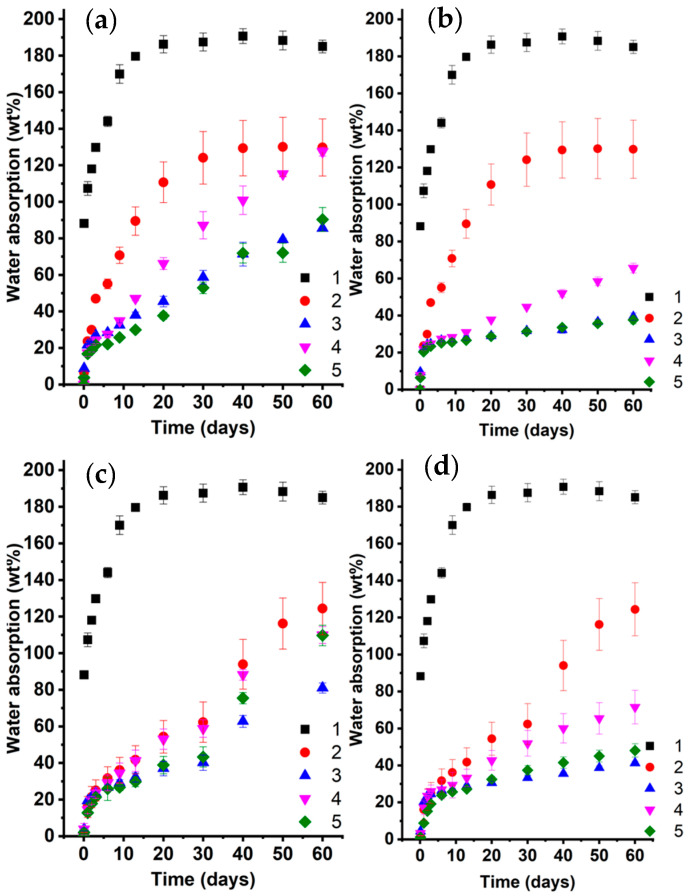
Water absorption of wood modified by immersion in epoxy varnish containing AlMA/FMA and GMA copolymers [copolymer content: (**a**) 3 wt.% and (**b**) 5 wt.%] and by brush application [copolymer content: (**c**) 3 wt.% and (**d**) 5 wt.%]: 1—unmodified wood; 2—base epoxy varnish; epoxy varnish with the addition of 3—poly(GMA-co-HFBMA), 4—poly(GMA-co-SMA), 5—poly(GMA-co-HFBMA-co-SMA).

The water-repellent properties of the modified wood were evaluated by monitoring time-dependent water absorption during prolonged immersion, taking into account both the application method and the type of hydrophobic modifier. The results show that using unmodified epoxy varnish as a protective coating reduces water absorption to approximately 130 wt.%. Notably, the coating application method considerably affects the rate of moisture uptake during the initial stages of the experiment. For samples prepared by immersion, water absorption reaches a plateau by day 30, approaching the maximum weight gain. In contrast, brush-applied coatings, which are thicker, exhibit a continuous increase in water uptake, reaching approximately 60 wt.% by day 30. The incorporation of FMA/AlMA and GMA copolymers considerably reduces water absorption (Figure 5a). As shown in Figure 5, increasing the hydrophobic modifier content from 3 to 5 wt.% decreases water absorption by more than twofold after 60 days of immersion, irrespective of the modifier type or coating application method. At the same time, samples modified with epoxy varnish containing poly(GMA-co-HFBMA) exhibited the highest water resistance. With a water absorption of 39 wt.% after 60 days of immersion, this composition is over three times more stable than the coating based on the unmodified epoxy varnish. In this study, we also synthesized ternary copolymers as hydrophobic modifiers, which ensure compatibility with epoxy varnish owing to the presence of SMA. Water absorption tests demonstrate that these modifiers perform well, comparable to the poly(GMA-co-HFBMA) copolymer, while also eliminating the need for a mixed solvent to dissolve the copolymer.

**Figure 6:**
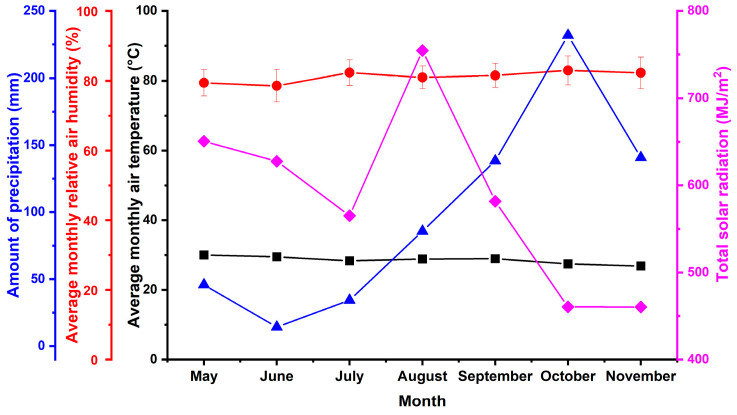
Meteorological data from the Dam Bai climatic testing station during the sample exposure period.

Recent advances in superhydrophobic materials and coatings focus on their practical application, with low mechanical durability being the primary limitation [54,55,56]. In this study, we have developed composite coatings that demonstrate effective water-repellent performance under laboratory conditions. To evaluate the performance of the water-repellent coatings under natural conditions, field tests were performed at the Joint Russian–Vietnamese Scientific Research and Technology Center’s climate station in a tropical marine environment at the Dam Bai Marine Research Station (Nha Trang Bay, Hon Tre Island, Vietnam). The harsh conditions of South Vietnam’s tropical climate provide a rigorous assessment of the durability and performance of polymer-based coatings. Real-world exposure tests are particularly valuable because they involve the simultaneous action of multiple physical and chemical factors with varying intensity and duration, which cannot be fully replicated under laboratory conditions [57]. Meteorological data recorded during the exposure period (Figure 6) indicate that the main aging phase occurred under high average air temperatures during the wet season. Additionally, the region experiences high levels of solar radiation, which can considerably influence the aging and degradation of polymer materials.

Unmodified wood and wood coated with unmodified epoxy varnish were used as controls. Exposure of unmodified wood led to material degradation, with surface hydrophilization observed after one month, followed by complete wetting. Wood samples coated only with epoxy varnish gradually lost their highly hydrophobic properties over six months, with contact angles decreasing from 120° to 78° for immersion-applied coatings and from 100° to 68° for brush-applied coatings. As shown in Table 4, the addition of copolymers to the epoxy varnish increases the stability of the hydrophobic properties and substantially reduces coating degradation. Based on contact angle measurements, immersion-applied coatings containing poly(GMA-co-HFBMA) exhibit the best performance, with contact angles decreasing to 144° after six months of exposure. However, despite maintaining high contact angles, the most critical parameter is the rate at which the contact angles change. Based on this criterion, the addition of poly(GMA-co-HFBMA-co-SMA) stands out. The enhanced stability of these coatings arises from the compatibility of the fluorinated hydrophobic copolymer with the epoxy varnish, creating a synergistic effect that combines the passive protection provided by the varnish film with the active reduction in surface free energy by the additives. Attention should also be given to changes in wood appearance resulting from exposure (photographs of samples are provided in the Supplementary Materials). Uncoated wood exhibits surface darkening after three months, and by six months, irreversible discoloration and surface desiccation occur, accompanied by crack formation. Coatings based on unmodified epoxy varnish offer some protection, but after six months of climatic exposure, surface darkening is still observed. Brush-applied coatings demonstrate less discoloration owing to the formation of a thicker and more uniform film. It is important to note that hydrophobic polymer additives not only enhance the stability of water-repellent properties but also help maintain the appearance of the coatings over time. After six months of exposure in a tropical climate, all composite coatings showed only slight surface darkening.

## 4. Conclusions

In summary, modifying pine wood with composite formulations based on industrial epoxy varnish and reactive copolymers of GMA and AlMA/FMA produces highly and superhydrophobic surfaces, with initial contact angles reaching up to 155°, while preserving the wood’s natural appearance. The method of application considerably influences coating properties: brush application smooths the original microtexture of the wood surface, whereas immersion preserves the original microtexture. The highest water-repellent performance is obtained using the functional poly(GMA-co-HFBMA) copolymer as an additive. Coatings formed by immersion of wood samples in an epoxy varnish containing poly(GMA-co-HFBMA) exhibit superhydrophobic behavior, with contact angles up to 155°. In contrast, brush-applied coatings achieve a highly hydrophobic state, with contact angles up to 138°. The incorporation of FMA/AlMA and GMA copolymers enhances the stability of the water-repellent properties. After 60 days of immersion in water, water absorption remains as low as 39 wt.%, more than three times lower than that of coatings based on unmodified epoxy varnish. Coatings incorporating ternary copolymer poly(GMA-co-HFBMA-co-SMA) have been shown to achieve a highly hydrophobic state, with contact angles reaching up to 138°, and to exhibit improved stability of their water-repellent properties. Climatic exposure tests under tropical conditions demonstrate that these functional copolymers not only improve the long-term stability of water-repellent properties—contact angles decrease to only 144° after six months—but also help preserve the visual appearance of the coatings.

## Figures and Tables

**Table 1:** Weight change in wood samples under different modification conditions with epoxy varnish-based composites.

Modifier	Concentration of Modifier Solution, wt.%	Change in Sample Mass Depending on Coating Method, wt.%	
		Immersion	Brushing
Epoxy varnish EP-2146	-	21.8 ± 4.4	20.9 ± 1.0
Poly-(GMA-co-HFBMA) *	3%	10.2 ± 1.0	17.4 ± 1.4
	5%	10.0 ± 1.0	13.0 ± 2.1
Poly-(GMA-co-SMA)	3%	19.3 ± 2.6	19.6 ± 2.5
	5%	17.2 ± 0.8	19.4 ± 1.4
Poly-(GMA-co-HFBMA-co-SMA)	3%	20.4 ± 2.0	17.8 ± 1.0
	5%	16.3 ± 0.3	17.5 ± 1.5

* Applied from a mixed solvent system (epoxy varnish: MEK).

**Table 2:** Chemical composition of wood sample surfaces under different modification conditions with epoxy varnish-based composites.

Modifier	Elemental Composition Depending on Coating Method, at.%					
	Immersion			Brushing		
	O	C	F	O	C	F
Unmodified wood ^1^	39.4	60.6	---	---	---	---
Epoxy varnish EP-2146	33.1	66.8	---	28.1	71.9	---
Poly(GMA-co-HFBMA) ^2^	31.0	64.17	4.8	28.9	67.6	3.5
Poly(GMA-co-SMA)	31.0	69.0	---	28.9	71.1	---
Poly(GMA-co-HFBMA-co-SMA)	30.7	68.3	1.0	28.2	70.7	1.1

^1^ Data for pre-washed and dried wood. ^2^ Product obtained from a mixed solvent (epoxy varnish: MEK).

**Table 3:** Initial contact angles on the surfaces of wood samples under different modification conditions with epoxy varnish-based composites.

Modifier	Measurement Surface	Contact Angle Depending on the Coating Method, °	
		Immersion	Brushing
The unmodified wood	perpendicular to fibers	121 ± 3	
	parallel to fibers	119 ± 2	
Epoxy varnish EP-2146	perpendicular to fibers	126 ± 3	100 ± 3
	parallel to fibers	104 ± 3	94 ± 4
Poly(GMA-co-HFBMA) *	perpendicular to fibers	152 ± 3	135 ± 3
	parallel to fibers	137 ± 3	124 ± 3
Poly(GMA-co-SMA)	perpendicular to fibers	129 ± 4	109 ± 2
	parallel to fibers	105 ± 3	95 ± 4
Poly(GMA-co-HFBMA-co-SMA)	perpendicular to fibers	135 ± 3	108 ± 2
	parallel to fibers	125 ± 2	102 ± 1

* Applied from a mixed solvent (epoxy varnish: MEK).

**Table 4:** Contact angles of wood samples under different modification conditions with epoxy varnish-based composites during exposure in a tropical climate.

Modifier	Measurement Surface	Contact Angle, °					
		Exposure Time, Month					
		0	1	2	4	5	6
The unmodified wood	perpendicular to fibers	121 ± 3	57 ± 5	wetted			
	parallel to fibers	119 ± 2	---	wetted			
Immersion							
Epoxy varnish EP-2146	perpendicular to fibers	120 ± 3	---	---	---	---	78 ± 2
	parallel to fibers	97 ± 3	---	---	---	---	72 ± 4
Poly(GMA-co-HFBMA) *	perpendicular to fibers	154 ± 3	141 ± 2	150 ± 2	135 ± 3	127 ± 4	144 ± 2
	parallel to fibers	140 ± 4	125 ± 2	124 ± 2	117 ± 2	109 ± 3	125 ± 1
Poly(GMA-co-SMA)	perpendicular to fibers	121 ± 2	130 ± 2	126 ± 3	121 ± 3	117 ± 2	112 ± 2
	parallel to fibers	98 ± 2	105 ± 3	91 ± 4	79 ± 1	78 ± 2	73 ± 3
Poly(GMA-co-HFBMA-co-SMA)	perpendicular to fibers	136 ± 3	136 ± 3	134 ± 2	136 ± 3	134 ± 2	134 ± 2
	parallel to fibers	119 ± 3	119 ± 2	119 ± 3	118 ± 3	117 ± 3	117 ± 3
Brushing							
Epoxy varnish EP-2146	perpendicular to fibers	100 ± 3	---	---	---	---	68 ± 7
	parallel to fibers	94 ± 4	---	---	---	---	62 ± 6
Poly(GMA-co-HFBMA) *	perpendicular to fibers	135 ± 3	140 ± 3	140 ± 4	143 ± 3	131 ± 2	119 ± 2
	parallel to fibers	124 ± 3	130 ± 3	130 ± 2	118 ± 4	108 ± 2	105 ± 2
Poly(GMA-co-SMA)	perpendicular to fibers	109 ± 2	104 ± 3	93 ± 2	91 ± 3	90 ± 3	89 ± 3
	parallel to fibers	95 ± 4	91 ± 2	86 ± 2	76 ± 1	73 ± 3	67 ± 2
Poly(GMA-co-HFBMA-co-SMA)	perpendicular to fibers	113 ± 2	106 ± 2	106 ± 3	108 ± 2	105 ± 2	105 ± 3
	parallel to fibers	108 ± 2	108 ± 2	108 ± 2	91 ± 1	86 ± 2	85 ± 5

* Applied from a mixed solvent (epoxy varnish: MEK).

## Data Availability

The original contributions presented in this study are included in the article/Supplementary Material. Further inquiries can be directed to the corresponding author.

## Supplementary Materials

The following supporting information can be downloaded at: https://www.mdpi.com/article/10.3390/polym17233172/s1, Figure S1: SEM images of defective areas on the surface of wood modified with epoxy varnish with the addition of poly-(GMA-co- HFBMA) (a) and poly-(GMA-co- HFBMA -co-SMA) (b) applied by brush; Figure S2: Images of the surface of unmodified wood exposed to tropical climate conditions; Figure S3: Images of wood surfaces modified with epoxy varnish by immersion (a) and brushing (b) after exposure to tropical climate conditions; Figure S4: Images of wood surfaces modified with epoxy varnish with additives: poly-(GMA-co- HFBMA) (application (a) by immersion and (b) by brush); poly-(GMA-co-SMA) (application (c) by immersion and (d) by brush); poly-(GMA-co- HFBMA -co-SMA) (application (e) by immersion and (f) by brush) after exposure to tropical climate conditions.
