# Supplementary material for: Hydrophobic Properties of Pine Wood Coatings Based on Epoxy Varnish and (Fluoro)Alkyl Methacrylate Copolymers
Source: Polymers (Basel). 2025 Nov 28;17(23):3172. doi: 10.3390/polym17233172 (PMC12694413; doi:10.3390/polym17233172)
Supplement: Supplementary file 1 [file polymers-17-03172-s001.zip › polymers-3961610-supplementary.pdf]

Hydrophobic Properties of Pine Wood Coatings Based on Epoxy Varnish and (Fluoro)Alkyl Methacrylate Copolymers

Viktor V. Klimov [1,*], Vladislav V. Arkhipov [1], Olga V. Klimova [1], Manh D. Le [2], Evgeny V. Bryuzgin [1] and Alexander V. Navrotskii [1]

[1] Chemical Engineering Faculty, Volgograd State Technical University, 28 Lenin Ave, 400005 Volgograd, Russia; vladislav.arkhipov2013@gmail.com (V.V.A.); ollik86@mail.ru (O.V.K.); bryuzgin_e@mail.ru (E.V.B.); a-navrotskiy@yandex.ru (A.V.N.)

[2] Southern Branch of Joint Vietnam-Russia Tropical Science and Technology Research Center, 3, 3/2 Str., District Vuon Lai, Ho Chi Minh City 740300, Vietnam; ducmanh89@gmail.com

* Correspondence: vicklimov@gmail.com

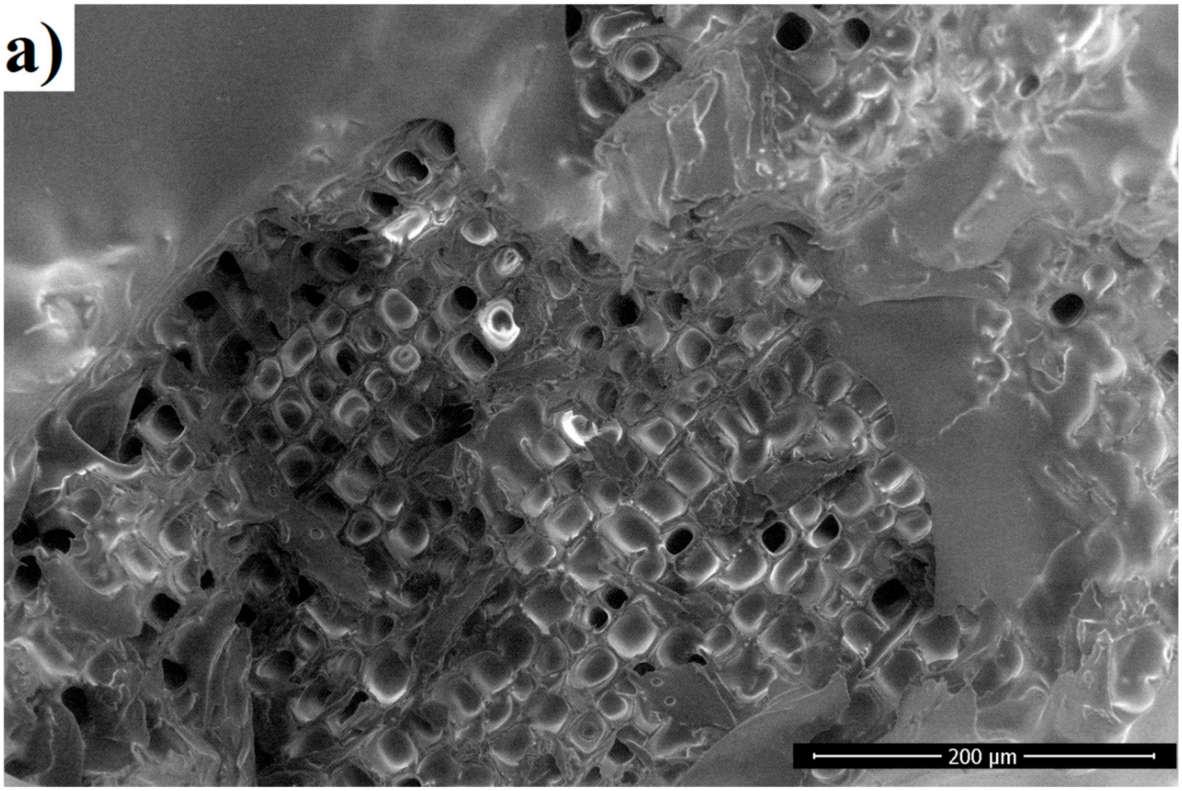

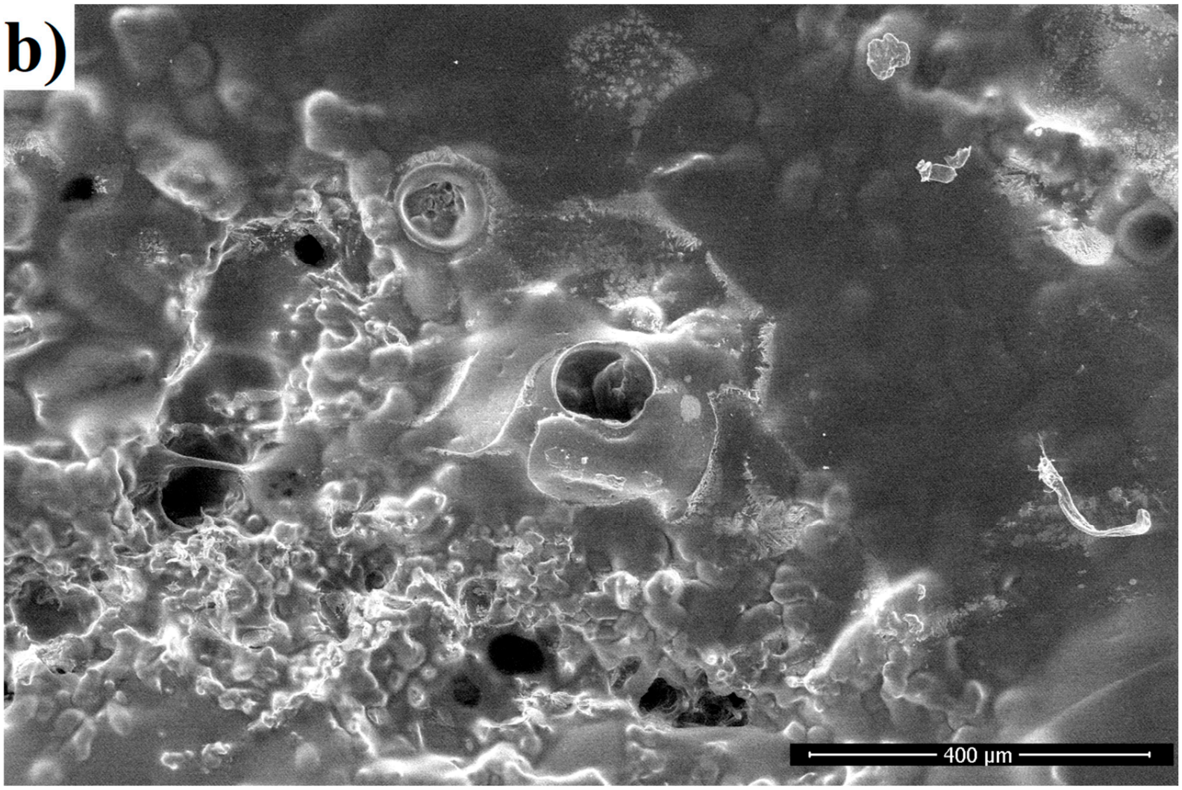

Figure S1 SEM images of defective areas on the surface of wood modified with epoxy varnish with the addition of poly-(GMA-co- HFBMA) (a) and poly-(GMA-co- HFBMA -co-SMA) (b) applied by brush

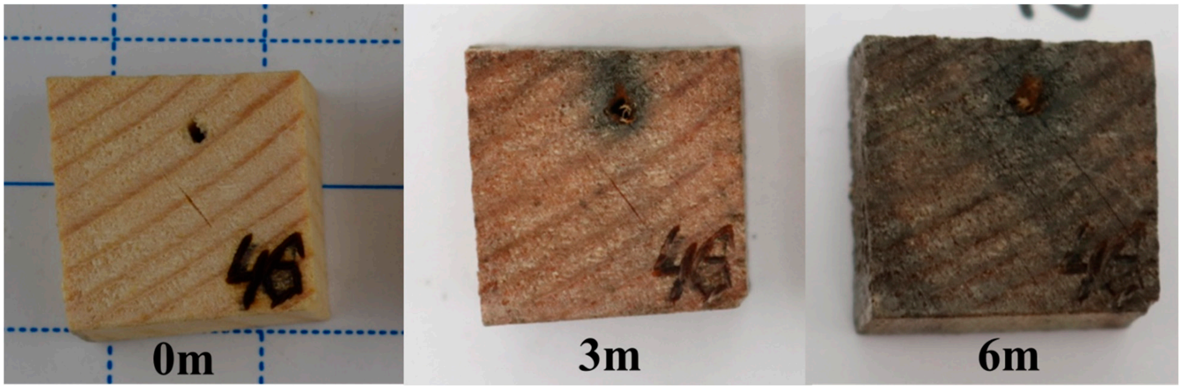

Figure S2 Images of the surface of unmodified wood exposed to tropical climate conditions

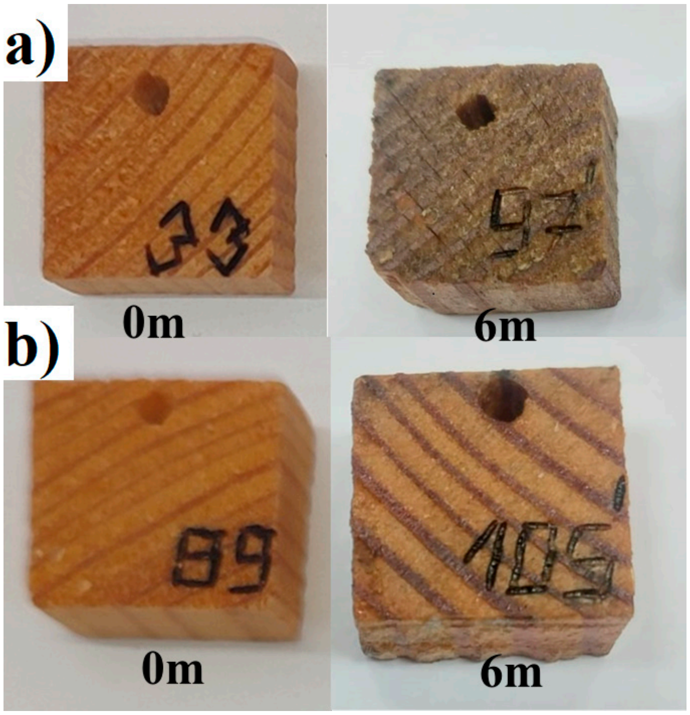

Figure S3 Images of wood surfaces modified with epoxy varnish by immersion (a) and brushing (b) after exposure to tropical climate conditions

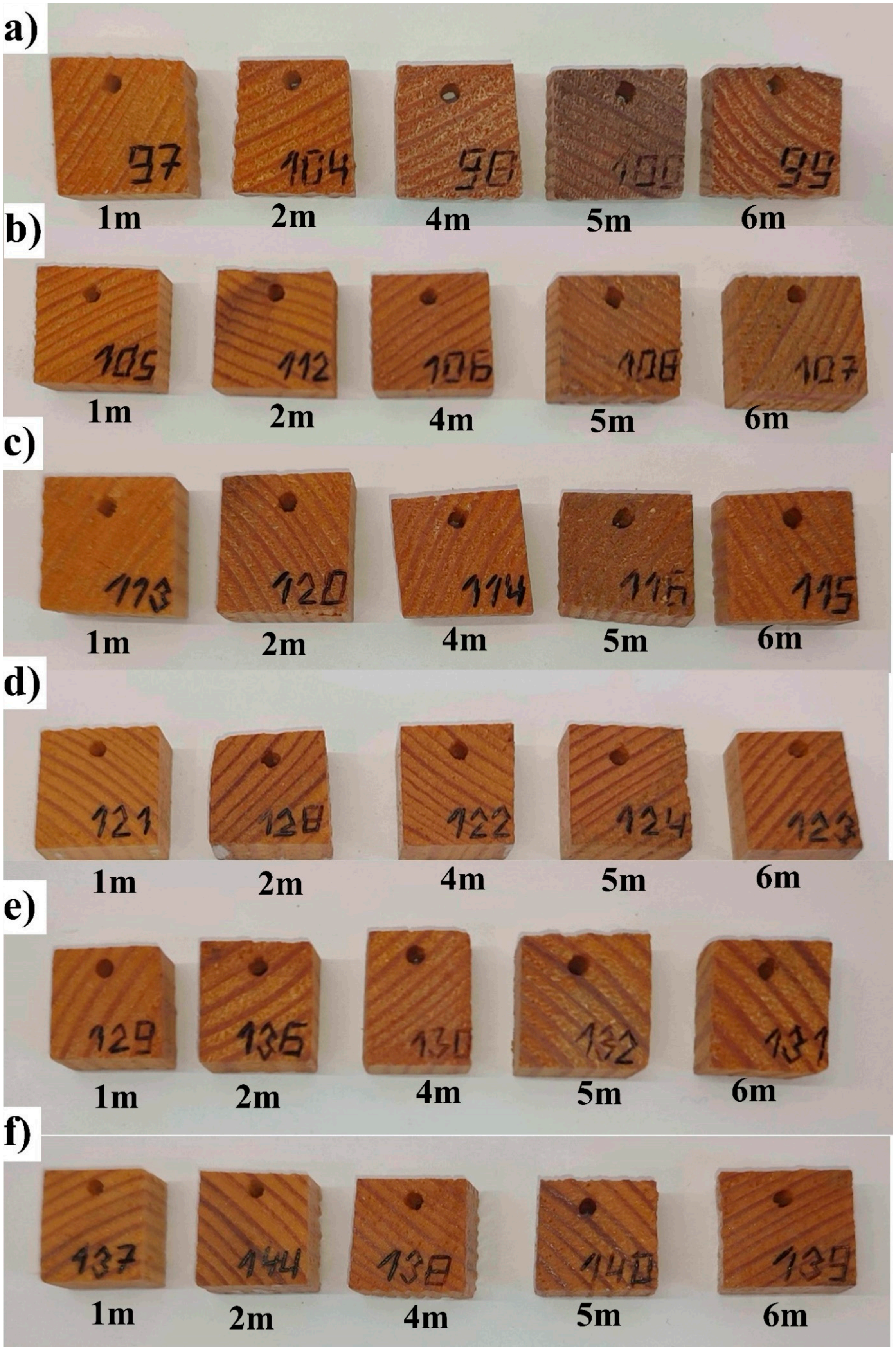

Figure S4 Images of wood surfaces modified with epoxy varnish with additives: poly-(GMA-co- HFBMA) (application (a) by immersion and (b) by brush); poly-(GMA-co-SMA) (application (c) by immersion and (d) by brush); poly-(GMA-co- HFBMA -co-SMA) (application (e) by immersion and (f) by brush) after exposure to tropical climate conditions
